# A Case of Tuberculosis, Supposed to Have Originated from Arsenious Acid Poisoning

**Published:** 1886-02

**Authors:** T. M. Holmes

**Affiliations:** Rome, Ga.


### A CASE OF TUBERCULOSIS SUPPOSED TO HAVE ORIGINATED FROM ARSENIOUS ACID POISONING.

BY T. M. HOLMES, M. D., ROME, GA.

READ BEFORE THE MEDICAL ASSOCIATION OF GEORGIA AT ITS THIRTY-SIXTH ANNUAL SESSION, SAVANNAH, APRIL 15, 1885.

This case which I have the pleasure of reporting is an illustration, I think, of an idea that I have for sometime entertained: that tuberculosis is sometimes produced by injury. That is to say, that tuberculosis may have its birth in an injured lung from whatever cause; that the injury may leave the lung in condition for the production of the micro-organism.

The case was in the person of a little negress about seven years old, living in a neighboring village. This patient was one of eleven, who, in the spring of 1883, were victims of arsenious acid poisoning. There were several families living in close proximity to each other, and in one of them was a wife and jealous husband. The latter had sworn to avenge himself of the grievance which he claimed his wife had committed against him of receiving the attentions of other men. For the purpose of vengeance, he brought home with him a sack of flower and some lager beer, in which he had previously placed arsenious acid. He invited his wife to partake of the beer, which she did, and requested that dinner be prepared from the flour for an absent working-man, whom it seems was one of the number whose lives he wished to take.

Soon after drinking the beer, the wife became very sick, and sent the flour to a neighbor to have it prepared for dinner. When it was ready for eating, quite a number ate of it, and all were made, in like manner, desperately sick, the sickness being characterized by severe griping in stomach and bowels, and vomiting and purging of the characteristic rice color. All the symptoms of arsenical poisoning were present.

When I arrived two of the victims were dead. With the assistance of two other physicians, I made an autopsy, and found echymoses of the mucous membrane of the stomach and bowels, the result of the poison. Taking the necessary precaution of tieing the viscera securely, I took them to the laboratory of Professor J. C. Lynes, the chemist of Shorter College. He made an analysis of the viscera and contents, and also of the dough and flour from which the bread was made, and in every instance found arsenic.

By prompt attention, the other victims, with one exception, made a final recovery—this exception being the subject of this paper.

Up to the time of poisoning, she was a strong and healthy child, but after this she never seemed to be her former self.

Her mother and father and maternal and paternal grandparents all had good histories. None of them, according to the evidence of the mother, died of consumption. A few weeks after she was poisoned, I was called to see her, and found her with high fever, a hacking cough, difficult breathing, great emaciation, feeble pulse, constipated bowels, no appetite and digestion bad.

She was put on a tonic course of treatment, and simple cough mixtures were given her. She only lived a few days after my second call. With the assistance of Drs. H. H. Battey, G. R. West and Mr. Geo. Glover, a medical student, I made a *post-mortem.*

The liver was the first viscera coming under our observation. It was noticed to be of a very peculiar appearance. It was literally covered with little white shining specks, and further exploration revealed the fact that the kidneys, spleen, lungs and intestines were of the same general appearance. We felt certain that this condition was the fatty degeneration anticipated as the result of the arsenical poisoning. Closer examination revealed thousands of miliary tubercles of a cheezy consistency. Could it be a case of sporadic or primary tuberculosis, or was it indeed the result of the introduction into the circulation of this poison?

The former, to say the least, is possible, but is not the latter, with the evidence in the case, more probable ? This may seem to be a far-fetched idea, but it is not an unusual occurrence for a case of phthisis to date from an injury, as the kick of a horse received on the chest, the patient having been previously strong and healthy.

Koch, the great discoverer of the bacillus tuberculosis, claims that there is no distinction between phthisis and tuberculosis only in degree or quantitatively. In the former, he claims that only one or a few of the pathogenic organisms find their way into the lungs. In phthisis, owing to the paucity of the bacillus infection, the patient may linger for months, or even years, while the deadly work is going on; but in tuberculosis there are myriads of these. Hence the term galloping consumption.

The principle is the same in either case, and may be compared to a race of a tortoise and a hare—the finale being the same. He also claims for consumption a specific germ, and that this germ finds a more congenial clime in some individuals than in others; that owing to certain predispositions of their physical constitution, there is in them a more favorable nurture ground; but while this is undoubtedly true, is it not a very plausible position to assume, that when the lungs, or other organs of the body, are inflamed from catarrh, traumatism or any other irritating agent, these swarming, invisible creatures may make an immediate onset upon these fresh fields in dependent state, doing their work of destruction and gaining a strong foot-hold ere nature has time to repair the breach? In this manner, I have no doubt that many cases of tuberculosis originate, and clinging to an old idea, long cherished, that one cannot have consumption without a naturally weak condition of the organs attacked, we say, "he was of a consumptive diathesis."

The idea of "diathesis" has been a fact long established, and we see it verified in numbers of families, one member after another, in successive generations, dying of consumption; but to believe that no one, outside of this regular line of hereditary weakness, dies of the disease is, to say the least, extremely paradoxical to my mind. I believe that the susceptibility to tuberculosis may be induced in subjects by no means of a consumptive predisposition. In common parlance, we hear the expression, "a consumptive form," and such cases are usually easily detected. Even the laity recognize them; but who can say with certainty that "such and such a man will never have consumption—he is too stout and healthy—none of his family ever had it?" I have known quite a number of just such cases to contract it and die of it, and to say that such a man was undoubtedly of a consumptive diathesis, or he never would have died of it, is equivalent to saying the same thing in regard to measles, or small-pox, or malarial fever, or any other disease.

I have known a wife who had never had the least symptoms of consumption, and whose family history was good, to contract pneumonia, which became chronic, the cough lingered, hemorrhages occurred, the expectoration became purulent and emitted a disagreeable odor, and very characteristic of consumption, her health declined and she became emaciated, the hectic flush bloomed upon her cheeks as the morning rose, and her face was as expressionless as that of a doll. She died of pneumonic phthisis, but was it tubercular ? Were the micro-organisms present ; and if so, who would say that she was of a consumptive diathesis ?

The husband of this woman was very stout and healthy, and his family history was in like manner good, but while his wife was in the advanced stage of her affliction, he was kicked on the chest by a horse, and shortly thereafter he began coughing, and after a few weeks had hemorrhage. The same symptoms that presented themselves in his wife developed in him, and he was hastened to an untimely end. He claimed that he contracted the disease by sleeping with his wife, which was undoubtedly the case ; but did he do so by virtue of a "predisposition," or was the lung made susceptible by the kick of the horse, which had produced a subacute pneumonetis ? To my mind, the latter seems far more plausible. Again, we ask the question, was it tuberculosis ? Was this work of destruction and death due to the presence of micro-organisms ? If not, how can the transmission be accounted for, and how does the work of disintegration or consumption of tissue go on, unless it be a living germ, eating like vermin upon the living being ?

It is claimed by some of Koch's strongest adherents, and members of the German council of doctors in 1884, that in each tubercle is found the characteristic bacillus tuberculosis perfectly visible under the microscope, and from this it would seem that the tubercle is only the cell of these organisms, as the gall-nut is the nidus of the oak-worm. Or, perhaps, a more rational idea would be, that the irritating effect of these organisms is the production of the tubercle, and that this work goes on until the vital forces of the individual are extinct. Now, as to the cases mentioned, which I have used as typical illustrations of a class, I do not think it at all probable that they would ever have had consumption, had it not been for the previous depressions, primarily in the one case an irritating poison, in the other a catarrhal inflam mation of the lungs. In both instances the various viscera were put in a condition of inability to resist the attack of the micro-organisms. In the latter cases, no *post-mortems* were held, but from the latest investigations in regard to the germ theory, may we not safely infer that the bacilli were present? Mr. Bland Sutton's late paper on "Lung Diseases in Wild Animals," read before the London Pathological Society, sets forth the fact that in twelve cases of pneumonic phthisis he had found more bacilli than in cases of tuberculosis, and in the latter they were found "litterly in millions." These animals had been brought from the tropics, and it seems that the depressing influence of the English climate was too much for them; hence the results he discovered. Now, it is well understood that there is a vast deal of difference between pneumonic phthisis and what is typically called tuberculosis, but what I wish to call especial attention to is what I believe to be the active agents in the destruction of the organs involved, the bacillus tuberculosis, and the circumstances under which they attack these organs: In this process there seem to be three prominent features. 1, that these organisms exist in the air and perhaps also in the water and in the earth; 2, that there must be a favorable nurture-ground, which every one does not possess, else all who breathe an atmosphere or drink water in which they exist would have tuberculosis; 3, that this favorable "nurture-ground" may be congenital, a predisposition, or may be produced by some depressing influence, as pneumonia, catarrh or traumatism, or by any agent sufficiently depressing in its effect.

---

DISGUISING THE TASTE OF MEDICINE.—Dr. Squibb, in *Ephemeris*, suggests that the sense of taste may be sufficiently benumbed by taking a few mouthfuls of iced water, then taking the disagreeable remedy in a wine-glassful of iced water, and if necessary, a few mouthfuls of cold water after the dose, to disguise the taste of most medicines, so that they may be readily taken.

